# Supplementary material for: The QPLEX™ Plus Assay Kit for the Early Clinical Diagnosis of Alzheimer’s Disease
Source: Int J Mol Sci. 2023 Jul 5;24(13):11119. doi: 10.3390/ijms241311119 (PMC10342648; doi:10.3390/ijms241311119)
Supplement: Supplementary file 1 [file ijms-24-11119-s001.zip › ijms-2456215-supplementary.pdf]

Table S1. Results of *ANCOVA* for various factors when age and gender are set as covariates

(A)

Education	N	Mean	SE	95% CI		Different ($P < 0.05$)
(1) CN	71	11.285	0.537	10.231	12.339	
(2) SCD	275	11.639	0.265	11.120	12.159	(3)
(3) MCI	857	10.140	0.151	9.844	10.436	(2)
(4) AD	192	10.767	0.317	10.146	11.388	

(B)

Hypertension	N	Mean	SE	95% CI		Different ($P < 0.05$)
(1) CN	71	0.428	0.060	0.311	0.545	
(2) SCD	268	0.467	0.030	0.409	0.525	
(3) MCI	845	0.522	0.017	0.489	0.555	
(4) AD	191	0.490	0.035	0.421	0.559	

(C)

Diabetes	N	Mean	SE	95% CI		Different ($P < 0.05$)
(1) CN	71	0.192	0.050	0.094	0.290	
(2) SCD	268	0.187	0.025	0.139	0.236	
(3) MCI	846	0.232	0.014	0.204	0.260	
(4) AD	189	0.195	0.030	0.137	0.253	

(D)

Hyperlipidemia	N	Mean	SE	95% CI		Different ($P < 0.05$)
(1) CN	66	0.483	0.064	0.358	0.608	
(2) SCD	259	0.556	0.031	0.495	0.616	(4)
(3) MCI	809	0.474	0.018	0.439	0.509	
(4) AD	188	0.403	0.036	0.332	0.475	(2)

(E)

Stroke	N	Mean	SE	95% CI		Different ($P < 0.05$)
(1) CN	70	0.011	0.026	-0.041	0.062	
(2) SCD	267	0.058	0.013	0.033	0.084	
(3) MCI	842	0.045	0.007	0.030	0.059	
(4) AD	189	0.059	0.016	0.028	0.089	

(F)

Angina	N	Mean	SE	95% CI		Different ($P < 0.05$)
(1) CN	71	0.094	0.033	0.029	0.159	
(2) SCD	263	0.073	0.017	0.041	0.106	
(3) MCI	834	0.091	0.009	0.072	0.110	
(4) AD	188	0.038	0.020	-0.001	0.077	

(G)

Thyroid	N	Mean	SE	95% CI		Different ($P < 0.05$)
(1) CN	71	0.112	0.041	0.032	0.192	
(2) SCD	260	0.179	0.021	0.139	0.220	(4)
(3) MCI	841	0.131	0.012	0.108	0.153	
(4) AD	188	0.083	0.024	0.035	0.130	(2)

(H)

Surgical history	N	Mean	SE	95% CI		Different ($P < 0.05$)
(1) CN	69	0.629	0.060	0.512	0.746	
(2) SCD	267	0.677	0.029	0.619	0.735	
(3) MCI	830	0.626	0.017	0.593	0.659	
(4) AD	185	0.587	0.035	0.518	0.656	

(I)

Cancer	N	Mean	SE	95% CI		Different ($P < 0.05$)
(1) CN	71	0.082	0.039	0.006	0.159	
(2) SCD	270	0.123	0.019	0.085	0.162	
(3) MCI	847	0.119	0.011	0.097	0.140	
(4) AD	190	0.102	0.023	0.056	0.147	

(J)

Family history	N	Mean	SE	95% CI		Different ($P < 0.05$)
(1) CN	71	0.237	0.057	0.125	0.349	
(2) SCD	272	0.375	0.028	0.320	0.431	
(3) MCI	851	0.302	0.016	0.270	0.334	(4)
(4) AD	191	0.409	0.034	0.343	0.475	(3)

(K)

Drinking	N	Mean	SE	95% CI		Different ($P < 0.05$)
(1) CN	71	0.599	0.055	0.490	0.707	(4)
(2) SCD	271	0.463	0.027	0.409	0.517	
(3) MCI	851	0.451	0.016	0.420	0.482	
(4) AD	191	0.389	0.033	0.324	0.453	(1)

(L)

Smoking	N	Mean	SE	95% CI		Different ($P < 0.05$)
(1) CN	71	0.218	0.039	0.141	0.295	
(2) SCD	271	0.247	0.019	0.209	0.285	
(3) MCI	851	0.236	0.011	0.215	0.258	
(4) AD	191	0.191	0.023	0.145	0.236	

(M)

BMI	N	Mean	SE	95% CI		Different ($P < 0.05$)
(1) CN	32	23.338	0.574	22.211	24.464	
(2) SCD	264	24.000	0.199	23.609	24.390	(4)
(3) MCI	810	23.614	0.114	23.390	23.837	
(4) AD	188	23.004	0.236	22.541	23.466	(2)

(N)

Anxiety	N	Mean	SE	95% CI		Different ($P < 0.05$)
(1) CN	71	0.156	0.041	0.076	0.236	
(2) SCD	270	0.258	0.020	0.218	0.298	
(3) MCI	850	0.259	0.012	0.236	0.281	
(4) AD	189	0.225	0.024	0.177	0.272	

ANCOVA results for (A) education years, (B) hypertension, (C) diabetes, (D) hyperlipidemia, (E) stroke, (F) angina, (G) thyroid, (H) surgical history, (I) cancer, (J) family history of dementia, (K) drinking, (L) smoking, (M) BMI, and (N) anxiety. If the Bonferroni corrected *P*-value of *ANCOVA* was less than 0.05, it was indicated in the "Different (P <0.05)" column. *ANCOVA*, analysis of covariance; SE, standard error; CI, confidence interval; BMI, body mass index; CN, cognitively normal; SCD, subjective cognitive decline; MCI, mild cognitive impairment; AD, Alzheimer's disease.
